# Peroxiredoxin 4 Interacts With Domeless and Participates in Antibacterial Immune Response Through the JAK/STAT Pathway

**DOI:** 10.3389/fimmu.2022.907183

**Published:** 2022-05-26

**Authors:** Xiao-qin Ran, Lin Gao, Meng Yan, Cui-jie Kang

**Affiliations:** Shandong Provincial Key Laboratory of Animal Cells and Developmental Biology, School of Life Sciences, Shandong University, Qingdao, China

**Keywords:** peroxiredoxin 4, JAK/STAT, Domeless, antimicrobial peptide, *Vibrio anguillarum*, *Marsupenaeus japonicus*

## Abstract

The JAK/STAT pathway plays an important role in the development and immune responses of animals. In vertebrates, families of cytokines or growth factors act as activators of the JAK/STAT pathway; however, the activators for the JAK/STAT signaling pathway in arthropods are largely unknown. Herein we report a new ligand, peroxiredoxin 4 (Prx4), for the Domeless in the JAK/STAT pathway of shrimp *Marsupenaeus japonicus*. Prx4 was induced to secrete into the extracellular surroundings upon *Vibrio* challenge, which then facilitated the anti-*Vibrio* activity of shrimp by activating the phosphorylation and nuclear translocation of STAT and the expression of STAT-responsive antimicrobial peptides. Blocking the expression of Prx4 *in vivo* abrogated the activation of the JAK/STAT pathway by *Vibrio* infection, while injection of Prx4 protein activated the pathway. The interaction between Prx4 and Domeless was proved by immuno-precipitation and protein pull-down assays. Moreover, two cysteine residues in Prx4 that are critical for the interaction and Prx4’s anti-*Vibrio* role were identified, and the binding site in Domeless for Prx4 was proved to be the cytokine-binding homology module fragment. Taken together, our study revealed a new function for Prx4 enzyme and established a new enzyme-type ligand for the activation of the JAK/STAT pathway in an aquatic arthropod.

## Introduction

The Janus kinase/signal transducer and activator of transcription (JAK/STAT) signaling pathway is involved in multiple physiological processes of animals, including embryonic segmentation, metabolism, and immune modulation (1–3). The canonical JAK/STAT signaling is activated by ligand binding to the transmembrane receptors; then, dimerization of the receptors is induced, and the intracellular domain of receptors is transphosphorylation by the associated intracellular JAK protein. The STAT protein is then phosphorylated and activated, which is translocated as a dimer into the nucleus to regulate target gene transcription (4). In vertebrates, more than 50 cytokines and growth factors are implicated in activating the JAK/STAT pathway through the membrane-located type I cytokine receptors, four intracellular JAKs JAKs (Jak1, Jak2, Jak3, and Tyk2) and seven STATs (STAT1, STAT2, STAT3, STAT4, STAT5a, STAT5b, and STAT6) have been reported (5, 6). In invertebrates, the components of the JAK/STAT pathway are conserved in several arthropod species, but they are simplified—for example, the complete components of the JAK/STAT pathway in *Drosophila* were identified, with only one JAK (encoded by *hopscotch* gene, *hop*), one STAT (encoded by *stat92E* gene or *Marelle*), three ligands (encoded by *unpaired* gene—*Upd1*, *Upd2*, and *Upd3*), one receptor (encoded by *Domeless* gene, *Dome*), and a short receptor (encoded by CG14225/*latran*) (7–11). In aquatic shrimp, the main components of the JAK/STAT pathway are also reported, including one *Domeless*, one *JAK*, and one *STAT*, and several antimicrobial peptides (AMPs) are identified as the effector genes of the pathway (12–16). Compared with mammals, little is known about the ligand that can activate the JAK/STAT pathway in aquatic arthropods. Moreover, in other invertebrate species, such as mollusks, nematodes, and echinoderms, only some components of the JAK/STAT pathway were reported, and whether or not they have a complete JAK/STAT pathway is still unclear (17–19).

Peroxiredoxins (Prxs) are a superfamily of antioxidases widely distributed in animals. The molecular size of these antioxidant enzymes ranges from 20 to 30 kDa. Based on the number of conserved cysteine residues and the action mode, six members of the Prxs family can be divided into three subtypes: typical 2-Cys (including Prx1, Prx2, Prx3, and Prx4 subtype), atypical 2-Cys (Prx5), and 1-Cys (Prx6) (20). In addition to acting as non-selenium-dependent peroxidases to catalyze the reduction of H_2_O_2_ and various organic hydroperoxides to form water and alcohols, the Prx members are also involved in signal regulation, apoptotic process, or tumor growth through hyperoxidation into sulfinic or sulfonic derivatives and/or various reversible modifications such as phosphorylation, glutathionylation, acetylation, nitrosylation, or hyperoxidation (21). The cellular localization, reaction mechanisms, and functions of Prx subtypes have been fully studied and reviewed in several articles (20, 22, 23).

In this study, we report a new function of shrimp Prx4 in promoting the anti-bacterial immune response of shrimp through activating the Domeless—Jak/stat signal pathway. The *in vivo* function of Prx4 in the anti-bacterial immunity of shrimp was investigated by survival assay and bacterial clearance assay, secretion of Prx4 in response to bacterial challenge was observed, and the activator activity of Prx4 to the JAK/STAT pathway was confirmed by antibody blocking and injection of exogenous Prx4 protein assays. The interaction of Prx4 with Domeless was also investigated by immunoprecipitation and protein pull-down assays. These results revealed a new model for the activation of Domeless—Jak/stat pathway in crustacean.

## Materials and Methods

### Immune Challenge and Tissue Collection

Healthy kuruma shrimp (*M. japonicus*) (about 9–12 g each) purchased from a seafood market in Qingdao, Shandong Province, were chosen as experimental materials. The shrimp was raised in laboratory conditions equipped with aquarium tanks with aerated seawater at 24°C for 3 days before infectious challenge and fed a commercial diet daily in the laboratory. For the bacteria challenge, each shrimp was injected with *Vibrio anguillarum* or *Staphylococcus aureus* (2 × 10^7^ CFU per shrimp), and phosphate-buffered saline (PBS) was injected as the control. For hemocyte collection, hemolymph was extracted from shrimp at different time points (2, 6, 12, 24, and 48 h) post-challenge (at least three shrimps at each time point) by using a syringe preloaded with ice-cold anticoagulants (0.45 M NaCl, 10 mM KCl, 10 mM EDTA, and 10 mM HEPES, pH 7.5) at a ratio of 1:1. Then, the hemocytes were collected by centrifuging the hemolymph sample at 800 × *g* for 10 min at 4°C. Gill tissue was collected simultaneously. Each sample was collected from at least five shrimp.

### RNA Extraction and cDNA Synthesis

Total RNA was extracted from the indicated tissues of shrimp using Trizol reagent (CWbio, Beijing, China) according to the manufacturer’s protocol. One hundred micrograms of tissues or 2 × 10^7^ cells were used for RNA extraction. The cDNA was synthesized using a Fast Quant First Strand cDNA Synthesis kit (Tiangen, Beijing, China) according to the manufacturer’s instructions.

### Quantitative Real-Time PCR

Quantitative real-time PCR (Q-PCR) was performed to determine the gene expression profiles. The experiment was performed according to the Ultra SYBR mixture protocol (with ROX, CWBio, Beijing, China) in a C1000 thermal cycler (Bio-Rad, USA) with the gene-specific primers listed in **Table 1**. The PCR procedure was as follows: 94°C for 5 min, 40 cycles of 94°C for 10 s, 60°C for 1 min, and a melting curve from 65 to 95°C. *β*-actin was used as the internal reference gene. The results were analyzed by the 2^-ΔΔCt^ method, and the data obtained were analyzed statistically using Student’s *t*-test and presented as mean ± SD. Significant differences were accepted at *p* < 0.05 (*) and *p* < 0.01 (**). All the experiments were repeated at least three times using individual templates.

**Table 1:** Sequences of the primers used in this study.

Primers	Sequence (5'-3')
**Gene cloning and recombinant expression**	
*Mj*STAT-exF	TACTCAGGATCCATGTCGTTGTGGAACAGAGC
*Mj*STAT-exR	TACTCAGGATCCATCAGCCGGCCAGC
*Mj*Prx4-exF	TACTCACTCGAGTGTACTGGTAACCAGTGATG
*Mj*Prx4-exR	TACTCACTCGAGCTGGTTGGCTTTCTTGA
*dmMj*Prx4exF	CACGGAGAGGTCTCCCCTGCCGGTTGG
*dmMj*Prx4exR	CCAACCGGCAGGGGAGACCTCTCCGTG
*MjDome1*-exF	TACTCAGAATTCTTGTTTCCCGCAACCATT
*MjDome1*-exR	TACTCACGCCGGCGGACCTGGAAACGACTTGG
*MjDome2*-exF	TACTCAGAATTCGTGAACAAAGTAGGCCGA
*MjDome2*-exR *MjDome1-1*-exF *MjDome1-1*-exR *MjDome1-2*-exF *MjDome1-2*-exR *MjDome1-3*-exF *MjDome1-3*-exR	TACTCACGCCGGCGATTCGTGGTCTCCTTCAA TACTCAGAATTCTTGTTTCCCGCAACCATT TACTCAGCGGCCGCCTGAACATCCTGTGGTGG TACTCAGAATTCGAGAGGAAAGTCAACATTGG TACTCAGCGGCCGCCACATTCTCATAAACACTGAT TACTCAGAATTCCCAAAACCAGCTGAGGAA TACTCAGCGGCCGCTGGAAACGACTTGGGAAC
**Quantitative Real-time PCR**	
*Mj*Prx4RTF *Mj*Prx4RTR *Mj*ActinRTF *Mj*ActinRTR	CTATGGGGTCTACCTGGAG TGCTGGTCTGTGAACTGG GCATCATTCTCCATGTCGTCCCAGT TACGGCTGCGAGAAGACGACAGAA
*MjAlfD1*RTF	GGCGAAAGGG AAATGTTTGGAC
*MjAlfD1*RTR	CCTTTCGCCGTTTTTCCACAA
*MjAlfA1*RTF	CGCTTCAAGGGTCGGATGCG
*MjAlfA1*RTR	CGAGCCTCTTCCTCCGTGATG
*MjAlfC1*RTF	CTGGTCGGTTTCCTGGTGGC
*MjAlfC1*RTR	CCAACCTGGGCACCACATACTG
*MjAlfC2*RTF	TCCTGGTGGTGGCAGTGGCT
*MjAlfC2*RTR	TGCGGGTCTCGGCTTCTCCT
*MjCrustinI-1*RTF	ATTCGCCCATAATCTTCC
*MjCrustinI-1*RTR	GCAGCACTTGTCGTCCC
*MjCrustinI-5*RTF	GTTGGCAGCCGTGTCTCG
*MjCrustinI-5*RTR	TGGGGTTGAATCTGGGTTTG
*MjDome*RTF	TGTTTCCCGCAACCATTA
*MjDome*RTR	TGATTCTTAGGGCAACGAGT
**RNA interference**	
DomeRNAiF	GCGTAATACGACTCACTATAGGCTGGGTATGTTGCTGTTGCG
DomeRNAiR	GCGTAATACGACTCACTATAGGGGGCAATGAATCCTGAGA
GFPRNAiF GFPRNAiR *Mj*Prx4RNAiF *Mj*Prx4RNAiR	GCGTAATACGACTCACTATAGGTGGTCCCAATTCTCGTGGAAC GCGTAATACGACTCACTATAGGCTTGAAGTTGACCTTGATGCC GCGTAATACGACTCACTATAGGATCAGCCGGCCAGCCCCAGAG GCGTAATACGACTCACTATAGGAGTATTTGAGCTTCTCCTCTG

### RNA Interference

The RNA interference (RNAi) was performed to detect gene function *in vivo* by injection of double-stranded RNA (dsRNA). The partial DNA fragment of indicated genes and the control gene (GFP) were amplified using primers (**Table 1**) linked to a T7 promoter. The PCR products were purified, enriched into 1 μg/μl, and utilized as the templates for dsRNA synthesis. The dsRNA was synthesized using T7 polymerase (Fermentas, USA) based on the method of Chen et al. (24). The RNAi assay was performed by injecting the specific dsRNAs (4 μg/g shrimp) into the shrimp hemocoel at the abdominal segment, and the control group was injected with an equal amount of control dsRNA. RNAi efficiency was determined using Q-PCR at 48 h after dsRNA injection.

### *In Vivo* Bacterial Clearance Assay

Bacterial clearance assays were performed to determine whether *Mj*Prx4 participated in inhibiting bacterial proliferation *in vivo*. The RNAi of *Mj*Prx4 or GFP was performed prior to the injection of *V. anguillarum* or *S. aureus* (3 × 10^7^ CFU). Hemolymph (200 μl) was collected from three shrimps of each group at 2 h post-injection of the bacteria and diluted 500 times with aseptic PBS. Diluted hemolymph (100 μl) was cultured on LB solid medium overnight at 37°C, and three repeats for each sample were performed. The number of bacterial colonies was counted. The assay was repeated twice. The final data of each sample were analyzed by GraphPad Prism software. The differences between experimental (*dsPrx4*) and control groups (*dsGFP*) were determined by *t*-test and indicated as *p* < 0.05 (*) and *p* < 0.01 (**).

### Expression and Purification of Recombinant Proteins and Preparation of Antibody

The cDNA sequence encoding the mature peptide of *Mj*Prx4, *Mj*STAT, *Mj*Dorsal, *Mj*Relish, or various fragments of *Mj*Dome was amplified using the specific primers listed in **Table 1** and ligated into the pET30a (+) or pGEX4T-1 plasmid. The recombinant vector was transformed into *Escherichia coli* for the expression of recombinant proteins after induction with 0.5 mM isopropyl-b-d-thiogalactopyranoside (IPTG). The inclusion bodies were extracted, washed, dissolved in buffer [0.1 mM Tris-HCl (pH 8), 10 mM DDT, and 8 M urea], and renatured by dialysis in PBS with 5% glycerol. The recombinant proteins were then purified using affinity chromatography with Ni-NTA Resin (TransGen Biotech) or GST-resin (GenScript, Nanjing, China). The endotoxins were removed by thoroughly washing the column with cold 0.1% Triton X-114 before the final elution of recombinant proteins from the column. The purified proteins were then dialyzed in PBS and stored at -80°C before use. A tag expressed by the empty vector was prepared simultaneously. The purified protein (5 mg) was sent to a company (Qingdao Kangda Biotechnology Company, Qingdao City, Shandong Province) for the preparation of polyclonal antibody in rabbit. The antiserum was collected 1 week after the second injection and stored at -80°C for use.

### Application of the Recombinant Proteins *In Vivo* and Antibody Blocking Assay

To confirm the function of *Mj*Prx4 in the activation of the JAK/STAT pathway, purified and endotoxin-free r*Mj*Prx4 or mutant r*Mj*Prx4 (3 µg/g shrimp) was injected into the shrimp. His-tag was used as the control. The hemocytes were extracted at 2 h after protein injection for immunocytochemistry detection, and the gills were collected at 2 or 6 h post-injection for Western blot detection. For antibody blocking experiments, antibody (IgG) was first purified from rabbit anti-*Mj*Prx4 serum or control serum. The serum was diluted into 20 mM Na_2_HPO_4_ (pH 8) and 0.15 M NaCl buffer, filtered through a 0.45-μm filter, and then loaded onto a Protein A column. The Protein A column was washed extensively with dilution buffer, and antibody (IgG) was eluted with 0.1 M glycine (pH 2.5), which were immediately neutralized by dialysis in 1/10 volume of 1 M Tris-HCl (pH 8.5) for 12 h and then in PBS at 4°C overnight. Purified antibody (IgG) was used to inject into the shrimp (3 µg/g shrimp).

### Protein Pull-Down and Co-immunoprecipitation Assays

GST pull-down assay was used to explore the possible interaction between *Mj*Prx4 and Dome *in vitro*. The recombinant vector pGEX-4T-1-*Mj*Prx4 and empty vector pGEX-4T-1 were transformed into *E. coli* Rosetta strain, respectively, for the expression of recombinant GST-Prx4 and GST proteins, and recombinant GST-Prx4 and GST were purified using affinity chromatography with GST-resin (GenScript, Nanjing, China). GST-Prx4 and GST proteins (20 μg each) were incubated with 20 μg His-tagged *Mj*Dome (His-*Mj*Dome), respectively, and the mixture was subjected to gentle agitation for 3 h at 4°C. GST resins (20 μl) were added to the mixture, and the agitation continued for another 1 h at 4°C. The resins were collected after centrifugation and washed three times with PBS. The bound proteins were eluted and analyzed by western blot using anti-GST or anti-His antibody.

The co-immunoprecipitation assay was performed to detect the secreted Prx4 in hemolymph and the interaction between Dome and Prx4 *in vivo*. The shrimp gills were homogenized in IP lysis buffer containing 50 mM EDTA, 50 mM Tris-HCl (pH 7.4), 150 mM NaCl, 1% Nonident-P40, and a protease inhibitor cocktail (Merck, Darmstadt, Germany; P8340). The homogenate was centrifuged at 12,000 × *g* for 10 min, and the resultant supernatant was precleared with Protein A Beads (GenScript; L00273) for 20 min and used as the protein pool of IP. Dome or Prx4 antiserum (30 μl) was incubated with 1 ml of the supernatant with gentle agitation for 3 h at 4°C. The Protein A beads were added to capture the interacting complex for 1 h with agitation at 4°C. After washing with PBS buffer, the immunoprecipitates were eluted by boiling the beads in SDS-PAGE sample buffer and detected by Western blotting.

### Western Blot

Western blot was performed to analyze the expression patterns of MjPrx4, MjDorsal, MjRelish, and MjSTAT, after the bacteria challenge. Shrimp tissues were thoroughly homogenized in RIPA lysis buffer (Beyotime, P0013), and the supernatant was collected for western blot. The separation of nuclear and cytoplasmic proteins was performed using a Nuclear Protein Extraction Kit (Sangon Biotech, Shanghai, China) according to the manufacturer’s instructions. The protein samples (20 µg) were separated by 10% or 12.5% SDS-PAGE and transferred onto a nitrocellulose membrane. The membrane was blocked with 3% skimmed milk in Tris-buffered saline for 1 h (TBS: 150 mM NaCl, 10 mM Tris-HCl, pH 7.5) and incubated with a specific primary antibody (1:300 dilution) at 4°C overnight. After washing three times by TBST (TBS containing 0.02% Tween-20), the membrane was incubated with Alkaline Phosphatase Goat anti-Rabbit IgG (H+L) (1:10000 dilution in blocking buffer, ZSGB Bio, Beijing, China) for 4 h. Finally, the membrane was washed by TBST, and protein band color was developed in a color-developing buffer (10 ml TBS containing 45 μl NBT and 35 μl BCIP). Western blot results were analyzed by software Quantity One and GraphPad Prism.

### Immunocytochemistry Assay

The hemolymph of shrimp was collected using a syringe containing 4% paraformaldehyde and anticoagulation mixture (1:1), and the hemocytes were then collected by centrifugation at 800 × *g* for 5 min at 4°C. After re-suspending in PBS, the hemocytes were dropped onto poly-lysine-coated glass slides and incubated for 1 h in a wet chamber. Then, washed with PBS and incubated in 0.2% Triton X-100 at 37°C for 5 min. After that, washed six times with PBS and blocked with 3% bovine serum albumin (dissolved in PBS) for 30 min at 37°C. The anti-*Mj*Prx4 or anti-*Mj*STAT antibody (1:400 dilution in 3% bovine serum albumin) was used as the primary antibody, and the goat anti-rabbit antibody conjugated with Alexa 488 (1:1,000 diluted in PBS) was used as the secondary antibody. The hemocytes were then stained with 4′,6-diamidino-2-phenylindole (DAPI) (1 µg/ml) for 10 min in the dark at room temperature. The excess DAPI was removed by washing six times with PBS, and the slides were examined under a fluorescent microscope (Olympus BX51, Japan). The colocalization percentage of Prx4 and DAPI-stained nuclei were analyzed using the Wright Cell Imaging Facility Image J software and compared with total cells (150 cells).

### Survival Rate Assay

A survival assay was performed to confirm the function of *Mj*Prx4 *in vivo*. The shrimps were randomly divided into three groups with 30 individuals in each group. Double-stranded RNA (dsRNA) of *Mj*Prx4 or GFP (control) was injected into the shrimp (3 μg dsRNA/g), and *V. anguillarum* infection (3 × 10^5^ CFU) was performed 24 h later by injecting the bacterium into the shrimp hemocoel. The number of surviving animals was monitored every 12 h for 4 days. The results were analyzed using the log-rank (Mantel–Cox) test in GraphPad Prism software.

## Results

### *Mj*Prx4 Promotes the Antibacterial Immune Response of Shrimp

**Figure 1:**
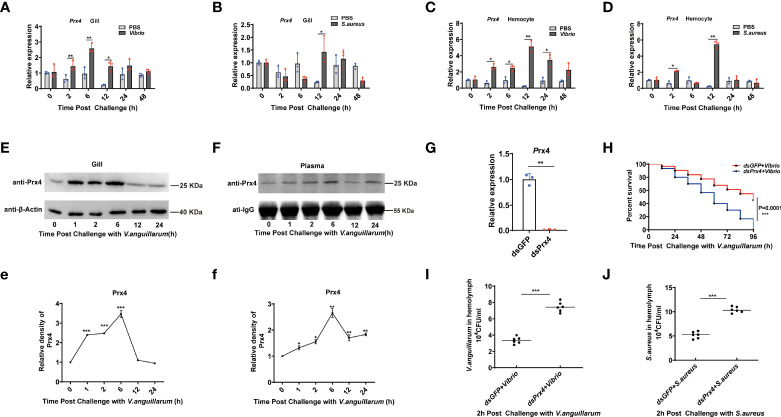
Expression pattern and function of *Mj*Prx4 in shrimp. **(A–D)** The expression patterns of *Mj*Prx4 in shrimp gills and hemocytes upon *V. anguillarum* or *S. aureus* infection were detected by Q-PCR. The significant differences between the bacteria-challenged samples and the phosphate-buffered-saline-injected samples were analyzed by paired Student’s *t*-test analysis and were marked by asterisks (*p < 0.05, **p < 0.01). **(E, F)** The protein expression patterns of *Mj*Prx4 in gills and hemolymph at different times after *V. anguillarum* infection. **(e**, **f)** The statistics of the experimental results in **(E**, **F)**. β-Actin and IgG were used as the internal control, and the results were analyzed by Student’s *t*-test for a significant difference. **(G)** Q-PCR detection of the knockdown specificity and efficiency of *Mj*Prx4 in shrimp. **(H)** Survival rate assay. *V. anguillarum* was injected into the shrimp after *Mj*Prx4 knockdown. The survival number was recorded every 12 h. The result was analyzed and shown by GraphPad Prism software. **(I, J)** The bacterial numbers in the hemolymph of shrimp were calculated in *Mj*Prx4 or GFP knockdown shrimp. The experimental data were analyzed using GraphPad Prism software. The significant differences between the two groups were accepted by Student’s *t*-test and indicated with asterisks (*p < 0.05, **p < 0.01, ***p < 0.001).

In our previous work, *Mj*Prx4 gene, a Prx4 subfamily member, was identified in the shrimp *Marsupenaeus japonicus*, and its function in the anti-virus immunity of shrimp was studied (24). To confirm whether *MjPrx4* was also involved in bacterial infection, the expression profiles of Prx4 after *S. aureus* and *V. anguillarum* infection were studied with Q-PCR. The results showed that, compared with the control group, a significant induction of the *MjPrx4* transcription level from 2 to 12 h post-infection with *V. anguillarum* was observed in both gills and hemocytes (**Figures 1A** **, C**). The transcription level of *Mj*Prx4 gene was also up-regulated by *V. anguillarum* infection in gills at 12 h and in hemocytes at 2 and 12 h post-infection (**Figures 1B** **, D**). These results indicated that *Mj*Prx4 mainly responds to gram-negative bacterial infection in the shrimp *M. japonicus*.

Furthermore, the expression patterns of *Mj*Prx4 protein in shrimp gills and hemolymph after *V. anguillarum* infection were examined. The results showed that the protein level of *Mj*Prx4 in gill tissue was significantly increased from 2 to 6 h after *V. anguillarum* infection and returned to normal levels at 12 h post-infection (**Figures 1E** **,e**). It is worth noting that the protein level of *Mj*Prx4 in hemolymph was also significantly increased 2 h after *V. anguillarum* infection, reaching the highest level at 6 h post-infection (**Figures 1F** **,f**). These results indicated that *V. anguillarum* infection induced the rapid translation of *Mj*Prx4 protein in shrimp tissues which were subsequently secreted into the extracellular environment. To analyze *in vivo* the potential immune function of *Mj*Prx4, we knocked down the *Mj*Prx4 expression by RNAi. As shown in **Figure 1G**, the *Mj*Prx4 transcriptional expression was successfully inhibited by dsRNA injection. The knockdown expression of *Mj*Prx4 led to an increase in the bacterial number in shrimp compared with the control group (**Figures 1I** **, J**) and a significant decrease in the survival rate (**Figure 1H**). Taken together, these results suggested that *Mj*Prx4 plays an important role in restricting bacterial infection.

### *Mj*Prx4 Functions in Anti-*Vibrio* Infection Through the JAK/STAT–AMP Pathway

**Figure 2:**
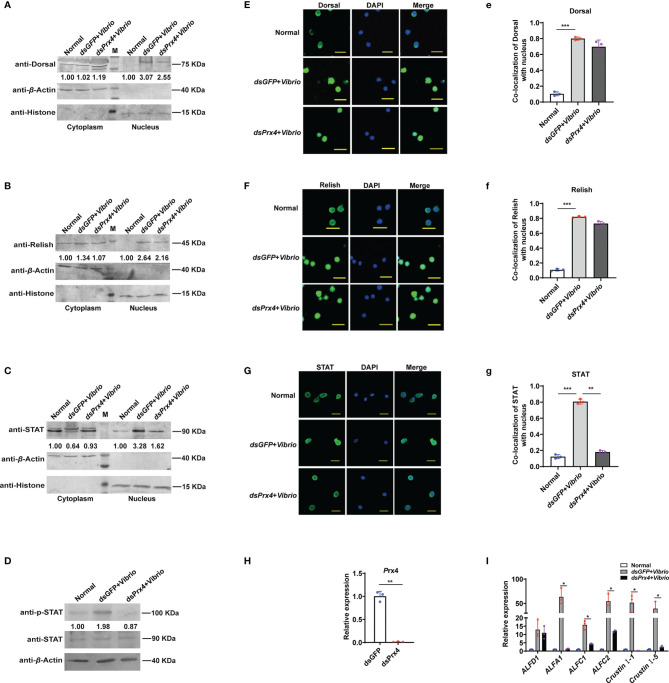
*Mj*Prx4 participated in anti-*Vibrio* immune response by promoting the JAK/STAT–AMP pathway in shrimp. **(A–C)** The nuclear translocation of Dorsal, Relish, and STAT in shrimp gills after *Mj*Prx4 RNAi and *V. anguillarum* infection was detected by Western blot, *dsGFP* injection was used as the control, β-Actin and Histone were used as internal controls for the cytoplasm and nuclear proteins. **(D)** Western blot analysis of the phosphorylation level of STAT in *Mj*Prx4-silenced shrimp and the control group. **(E–G)** Immunocytochemistry detects the nuclear translocation of the transcription factors Dorsal, Relish, and STAT in shrimp hemocytes. The *dsGFP*-treated group was used as the control. Scale = 10 µm. **(e–g)** Statistical analysis of the co-localization of Dorsal, Relish, and STAT with the nucleus in **(E–G)** using WCIF Image J software. **(H)** The knockdown specificity and the efficiency of *Mj*Prx4 in shrimp were analyzed by Q-PCR. β-Actin was used as the internal control. **(I)** The expression of antimicrobial peptides downstream of STAT in *Mj*Prx4-silenced shrimp was detected by Q-PCR. The experimental data were analyzed using GraphPad Prism software. The significant differences between the two groups were accepted by Student’s *t*-test and indicated with asterisks (*p < 0.05, **p < 0.01, ***p < 0.001).

In shrimp, three signaling pathways, Toll, IMD, and JAK/STAT, were proved to be functional in anti-*V. anguillarum* immune response *via* promoting the expression of specific antimicrobial peptides (16, 25–27), and obvious nuclear translocation of the transcription factors (Dorsal, Relish, and STAT) were seen at the early time of *V. anguillarum* infection. To explore the possible immune signaling pathways that *Mj*Prx4 may be involved in, RNAi was performed, and the nuclear translocation of the three transcription factors—Dorsal, Relish, and STAT—was detected at 6 h after *V. anguillarum* challenge. The specific polyclonal antibodies for the transcription factors Dorsal, Relish, and STAT were used for detection, and their specificity to the antigen in shrimp tissues was detected and is shown in **Supplementary Figure 1**. The Western blot analysis of nuclear and cytoplasmic separation showed that the nuclear translocation of the transcription factors (Dorsal, Relish, and STAT) was increased upon *Vibrio* challenge (*ds*GFP + *Vibrio* group *vs*. normal group). In contrast, the nuclear translocation of STAT, neither Dorsal nor Relish, was decreased in *Mj*Prx4-silenced shrimp (*dsPrx4* + *Vibrio* group *vs*. *dsGFP* + *Vibrio* group) (**Figures 2A** **–C**). Furthermore, the phosphorylation of STAT was detected and showed a depression in *Mj*Prx4-silenced shrimp compared with the control group (*dsGFP* + *Vibrio* group) (**Figure 2D**). The immunocytochemical analysis of hemocytes from *Mj*Prx4-silenced shrimp and control shrimp yielded similar results (**Figures 2E** **–G**), indicating that *MjPrx4* is an epistatic gene to the JAK/STAT pathway upon *V. anguillarum* infection.

To further confirm the results, the expression pattern of several antimicrobial peptide (AMP) genes (GenBank accession numbers *ALFA1* KU213607, *ALF-C1* KU213608, *ALF-C2* KU160498, *ALF-D1* KU160499, *CruI-1* KU160502, and *CruI-5* KU213606) was studied, and these AMP genes were reported as the effector genes downstream of the JAK/STAT pathway during *V. anguillarum* in *M. japonicus* in a previous research (16). As shown in **Figures 2H, I**, the induced expression of five JAK/STAT-responsive AMP genes (*ALFA1*, *ALF-C1*, *ALFC2*, *Cru I-1*, and *Cru I-5* in the *dsGFP* + *Vibrio* group) were inhibited in the *dsPrx4* + *Vibrio* shrimp, and this inhibition was specific to the effector genes downstream of the JAK/STAT pathway since the expression of the control AMP gene (*ALFD1*) was not changed. Taken together, the above-mentioned results showed that *Mj*Prx4 promoted the anti-*Vibrio* immunity of shrimp through the JAK/STAT pathway.

### Activation of the JAK/STAT–AMP Pathway Depends on Extracellular *Mj*Prx4

**Figure 3:**
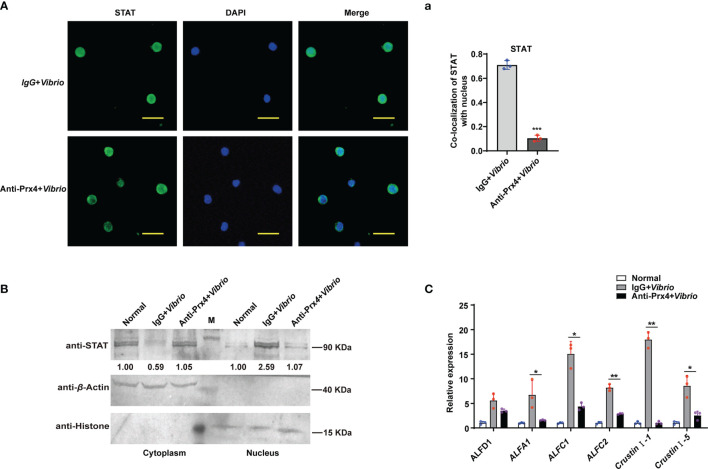
Antibody blocking of the extracellular *Mj*Prx4 inhibited the nuclear translocation of STAT. **(A)** *V. anguillarum* challenge was performed 0.5 h after the antibody of *Mj*Prx4 was injected into the shrimp. Preimmune IgG was used as the control, and the cellular distribution of STAT was detected by immunocytochemistry. The scale is 10 μm. **(a)** The statistical analysis of **(A)** by using WCIF Image J software. **(B)** The subcellular distribution of STAT in gills was detected by Western blot. β-Actin and histone H3 were used as loading controls for the cytoplasmic and nuclear proteins, respectively. **(C)** The expression of antimicrobial peptide genes was detected by Q-PCR. β-Actin was used as the control. The experimental data were analyzed using GraphPad Prism software. The data were analyzed by Student’s *t*-test, and significant differences between the two groups were accepted and indicated with asterisks (*p < 0.05, **p < 0.01, ***p < 0.001).

Since *Mj*Prx4 was secreted extracellularly during *V. anguillarum* infection in shrimp (**Figure 1F**), to reveal whether the activation of the JAK/STAT–AMP pathway upon *Vibrio* infection was due to the extracellular *Mj*Prx4 or by the intracellular form, the antibody-blocking assay was performed. The specific antibodies of *Mj*Prx4 or control IgG were injected into the shrimp to block the extracellular *Mj*Prx4, and then the *Vibrio* infection was performed. The nuclear translocation of STAT and the expression of AMPs in the shrimp tissues were detected subsequently. It is notable that the nuclear translocation of STAT in hemocytes was significantly reduced in the experimental group (anti-Prx4 + *Vibrio*) compared with the control group (IgG + *Vibrio*) (**Figures 3A** **,a**). Similarly, the nuclear translocation of STAT in gills was also significantly inhibited (**Figure 3B**). At the same time, the Q-PCR detection showed that the upregulated expression of JAK/STAT-responsive AMPs was also depressed by the *Mj*Prx4 antibody blocking (**Figure 3C**). These results indicated that the extracellular *Mj*Prx4 played an essential role in activating the JAK/STAT–AMP pathway in shrimp. It may function as a cytokine-like factor in the anti-*Vibrio* immune response of shrimp.

### Extracellular *Mj*Prx4 Acts as a Cytokine to Activate the STAT-AMP Pathway

**Figure 4:**
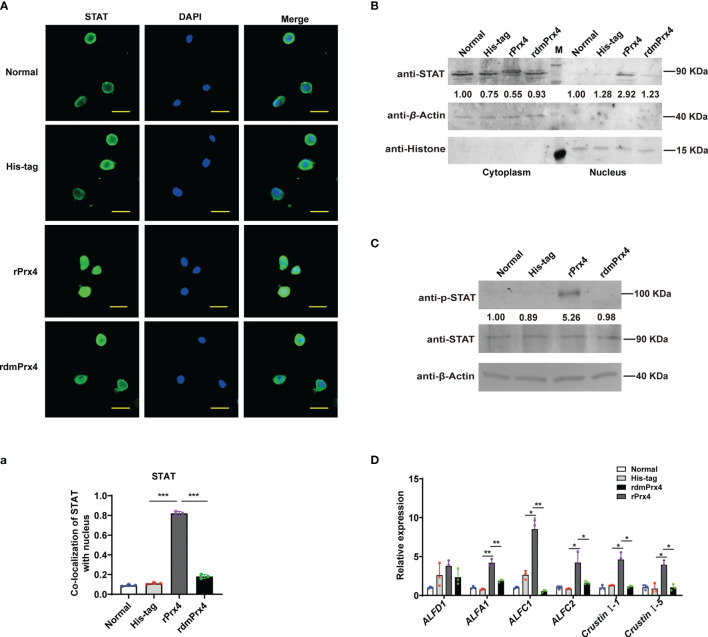
Wild-type *Mj*Prx4 induced STAT translocation into the nucleus. **(A)** Purified and endotoxin-free rMjPrx4 or mutants rMjPrx4 (3 µg/g shrimp) were injected into the shrimp, and STAT distribution in hemocytes was detected at 1 h post-injection. His-tag protein was used as the control; the scale is 10 μm. **(a)** The localization of STAT within the nucleus was analyzed using WCIF Image J software. Significant differences between the indicated two groups were accepted after Student’s *t*-test analysis and indicated with asterisks (***p < 0.001). **(B)** The subcellular distribution of STAT in gills was detected by Western blotting after recombinant protein injection. **(C)** Western blot analysis of the phosphorylation level of STAT in normal and recombinant protein injection shrimp. His-tag protein was used as the control. **(D)** The expression of antimicrobial peptide genes in the normal and recombinant protein injection shrimp was detected by Q-PCR. The experimental data were analyzed using GraphPad Prism software. The significant differences between the indicated two groups were accepted after Student’s *t*-test analysis and indicated with asterisks (*p < 0.05, **p < 0.01).

To further confirm whether extracellular *Mj*Prx4 possesses cytokine-like function to activate the JAK/STAT–AMP pathway, the inactive mutant *Mj*Prx4 protein (rdmPrx4, double mutant on cysteine 97 and cysteine 218) was prepared, and the antioxidant activity of purified wild-type rPrx4 or inactive mutant rdmPrx4 protein was detected and is shown in **Supplementary Figure 2**. Then, rPrx4 or rdmPrx4 protein was injected into the shrimp, and the nuclear translocation of STAT in gills and hemocytes was detected. The results showed that the injection of exogenous rPrx4 protein alone significantly promoted the nuclear translocation of STAT in gills and hemocytes, while the exogenous His-tag or dmPrx4 protein injection did not have an effect on STAT nuclear translocation compared with the normal control, indicating that only wild-type *Mj*Prx4 protein was efficient in activating the nuclear translocation of STAT (**Figures 4A** **,a, B**). Moreover, the phosphorylation of STAT in shrimp gills and the expression patterns of AMPs were also detected. The results showed that the rdmPrx4 or His-tag protein injection did not affect the phosphorylation of STAT and the expression of the STAT-responsive AMPs compared with the normal group. Unlike those two groups, the phosphorylation of STAT and the expression of five STAT-responsive AMPs (ALFA1, ALF-C1, ALFC2, Cru I-1, and Cru I-5) were both upregulated by rMjPrx4 injection (**Figures 4C** **, D**). These results indicated that the exogenous wild-type *Mj*Prx4 can act as a cytokine to activate the STAT-AMP pathway *in vivo*.

### *Mj*Prx4 Regulates the JAK/STAT Pathway Through the Domeless Receptor

**Figure 5:**
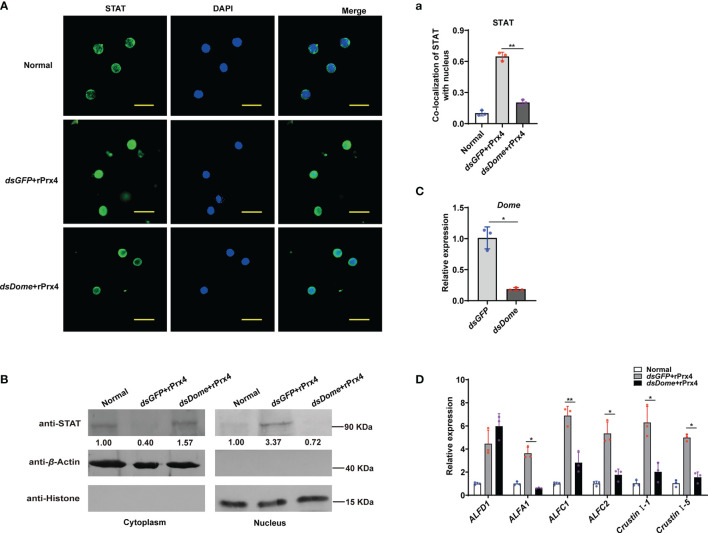
Extracellular *Mj*Prx4 promoted the activation of the JAK/STAT pathway through the Domeless receptor. **(A)** The subcellular location of STAT in hemocytes of Dome-silenced shrimp or the *ds*GFP control group was detected by immunocytochemistry. r*Mj*Prx4 (3 µg/g) was injected into the shrimp 24h after the injection of dsDome or dsGFP. **(a)** The localization of STAT in the nucleus was calculated by WCIF Image J software. Significant differences between the indicated two groups were shown as **p < 0.01. **(B)** Western blot detection of the subcellular distribution of STAT in gills. **(C)** The RNAi efficiency of Dome gene was detected by Q-PCR. Significant differences between the indicated two groups were analyzed by Student’s *t*-test and shown as **p < 0.01. **(D)** Expression patterns of antimicrobial peptide genes in shrimp gills after treatment as described in **(A)**. Three independent repeated experiments were performed, and Student’s *t*-test was used for significant difference analysis (*p < 0.05, **p < 0.01).

Domeless (Dome) was proved to be the upstream membrane receptor of the JAK/STAT pathway in the shrimp *M. japonicus* (16). To reveal the possible mechanism of *Mj*Prx4 in activating the JAK/STAT–AMP pathway, Dome expression was knocked down, and then rPrx4 was injected into the shrimp; the nuclear translocation of STAT in hemocytes and gills was detected. As shown in **Figure 5C**, the expression level of Dome was decreased significantly in the Dome-silenced shrimp. Compared with the *dsGFP* group, the nuclear import of STAT in the hemocytes caused by r*Mj*Prx4 injection was significantly reduced in the Dome-silenced group (**Figures 5A**,**a**). Similarly, the nuclear translocation of STAT in gills was also inhibited (**Figure 5B**). In addition, the expression of five STAT-responsive AMPs (*ALFA1*, *ALFC1*, *ALFC2*, *CruI-1*, and *CruI-5*) was significantly impaired, while the expression of *ALFD1* was not affected (**Figure 5D**). These results indicated that the expression of Dome was needed for the regulation function of extracellular *Mj*Prx4 to the JAK/STAT–AMP pathway.

### *MjPrx*4 Interacts With the CBM Domain of Domeless

**Figure 6:**
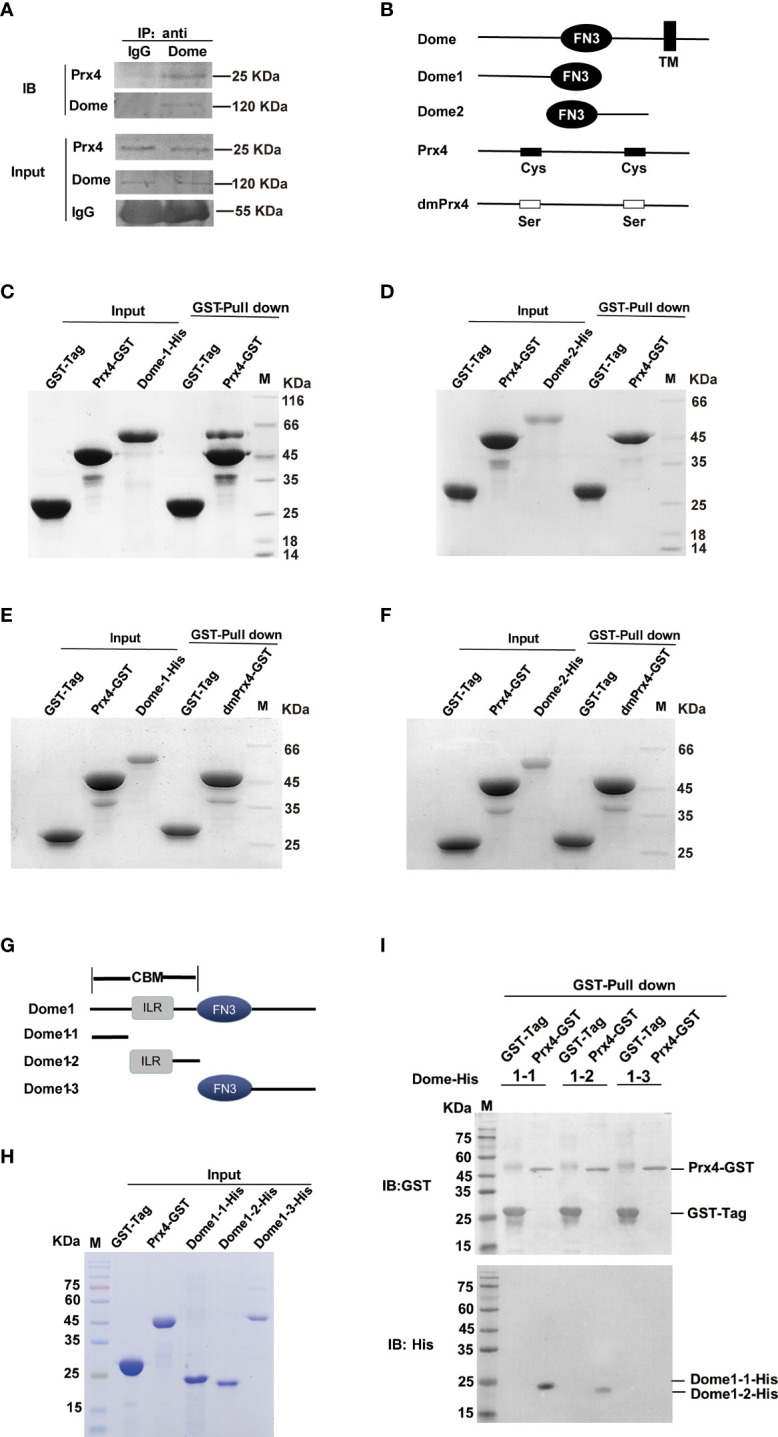
*Mj*Prx4 interacted with Dome. **(A)** Co-immunoprecipitation was performed to detect the interaction between *Mj*Prx4 and *Mj*Dome in the gills of shrimp. **(B)** Sketch map for the *Mj*Dome and *Mj*Prx4 mutant proteins used in this study. Mutant Dome1 (19–457 amino acids) and Dome2 (424–846 amino acids) in the extracellular segment of wild-type Dome were expressed as His-tagged recombinant proteins. The two crucial cysteine residues (Cys97 and Cys218) of *Mj*Prx4 were mutated into serine to generate mutant *Mj*Prx4 (dmPrx4). **(C, D)** GST pull-down assay was performed to detect the interaction of rPrx4-GST with rDome1-His or *r*Dome2-His. **(E, F)** GST pull-down assay was performed to detect the interaction of rdmPrx4-GST with rDome1-His or *r*Dome2-His. **(G)** Sketch map for Dome1 (19–457 amino acids) mutant protein used in this study. Three fragments of Dome1 (19–457 amino acids) were generated and named Dome1-1 (19–139 amino acids), Dome1-2 (125–221 amino acids), and Dome1-3 (222–456 amino acids). **(H)** The purified GST-tag, rPrx4-GST, Dome1-1-His, Dome1-2-His, and Dome1-3-His protein for pull-down assay were detected by SDS-PAGE. **(I)** GST pull-down assay was performed to detect the interaction of *Mj*Prx4 with *Mj*Dome1-1, *Mj*Dome1-2, or *Mj*Dome1-3. The bound proteins were eluted and analyzed by Western blot using the GST or His antibody.

To confirm whether *Mj*Prx4 interacts with *Mj*Dome, *in vivo* immunoprecipitation and *in vitro* protein pull-down assay were performed. Gills from *V. anguillarum*-infected shrimp were collected for the endogenous immunoprecipitation of Prx4 with Dome. IgG from preimmune rabbit was used as the control, and then western blot analysis was performed by using anti-Prx4 and anti-Dome antibodies. **Figure 6A** shows that *Mj*Prx4 can interact with *Mj*Dome *in vivo*. Shrimp Dome (GenBank accession no. KX358405) comprises a signal peptide, a cytokine-binding homology module region (CBM) with an interleukin 6 receptor (ILR) alpha domain inside, five fibronectin type-III (FN3) domains, and a transmembrane (TM) region (**Supplementary Figure 3**). Then, two His-Tag-truncated fragments of Dome protein, Dome1 (including the CBM region plus two FN3 domains) and Dome2 (from the fourth FN3 domain to the TM region), as shown in **Figure 6B**, were recombinantly expressed in *E. coli* system, and the interaction of truncated Dome proteins with GST-*Mj*Prx4 was studied by protein pull-down assay. The results showed that *Mj*Prx4 could interact with truncated Dome1, but not with Dome 2 (**Figures 6C** **, D**), and the mutant rdmPrx4 protein did not interact with Dome1 or Dome2 (**Figures 6E** **, F**). These results suggest that *Mj*Prx4 may interact with the CBM region in the Dome receptor.

To further disclose the interaction of Dome with *Mj*Prx4, three truncated Dome1 fragments were further prepared as shown in **Figure 6G**. Dome1-1 included 19 to 139 amino acids of the CBM region, Dome1-2 contained the remainder CBM region with the IRL domain inside (125 to 221 amino acids), and the rest of the part of Dome1 was expressed as Dome1-3 (222 to 456 amino acids). As the molecular weights of Dome1-1 and Dome1-2 were similar and can not be distinguished by size (**Figure 6H**), the results of the pull-down assay were detected by Western blot. **Figure 6I** shows that the input GST-Prx4 protein or GST protein was at an almost equal amount; only the binding of Dome1-1 and Dome1-2 with GST-Prx4 was detected, and Dome1-3 did not interact with GST-Prx4. Taken together, these results suggest that *Mj*Prx4 interacts with the CBM region of *Mj*Dome, and the two cysteines of *Mj*Prx4 are necessary for the interaction.

## Discussion

**Figure 7:**
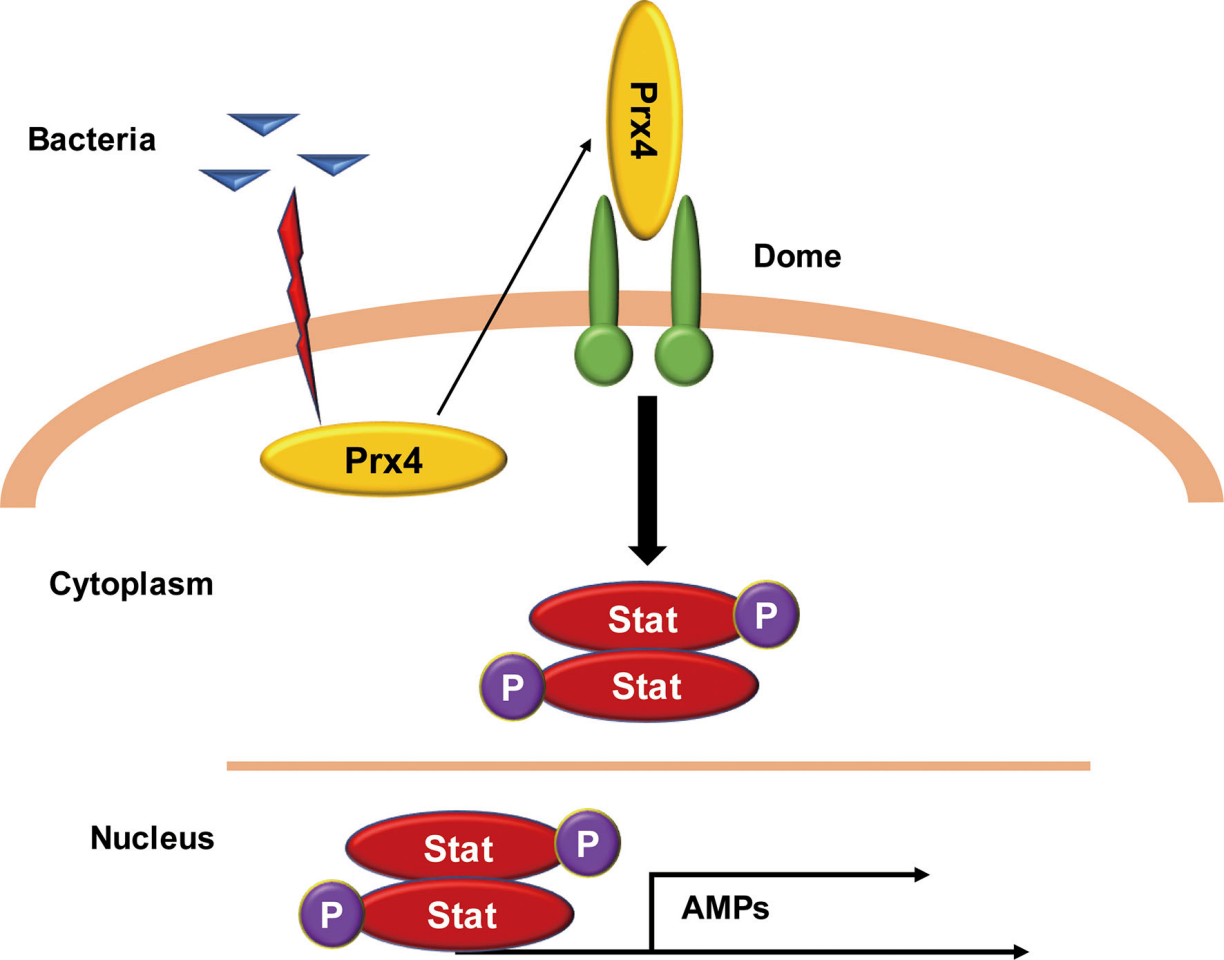
Model of the *Mj*Prx4-mediated anti-*Vibrio* mechanism. The intracellular *Mj*Prx4 is induced to secrete into hemolymph during *Vibrio* infection, which then binds to the cytokine-binding homology module region of the extracellular Domeless receptor, leading to the phosphorylation and nuclear translocation of STAT transcription factor that transcriptionally regulates the expression of AMPs and contributes to inhibit the *Vibrio* infection.

In this report, the secretion of active Prx4 protein into the hemolymph of shrimp was identified upon *Vibrio* challenge. Prx4, in turn, bound to the CBM region of the extracellular Domeless receptor, which resulted in activating the JAK/STAT signaling and induced the expression of the STAT-responsive AMPs, which overall contributed to the antibacterial immunity of the shrimp. The schematic diagram of this process is shown in **Figure 7**.

Among the Prx antioxidant enzyme superfamily in mammals, Prx1 and Prx4 are two known secretory forms. The cytosolic Prx1 can be released into the extracellular space under certain situations and then stimulate the TLR4-dependent secretion of TNFα and IL-6 from immune cells, indicating that Prx1 functions as the damage-associated molecular pattern (28). Differently from Prx1, a unique leader peptide for secretion is present in the N-terminus of Prx4 which confers its secretion. The activator activity of mammalian Prx4 (also known as TRANK) in immune signaling pathways has been reported (29). When Prx4 was overexpressed in insect cells, the secreted form of Prx4 was detected. The binding of Prx4 with heparin sulfate in the cell surface of endothelial cells was reported (29). In addition, exogenous Prx4 added to the human myeloid U-937 cells activated NF-κB in a dose- and time-dependent manner, which induced the degradation of the inhibitory subunit of NF-κB and also led to an increase in JNK activity in a time-dependent fashion (30). Although the mechanism for that phenomenon was not illustrated yet, a cytokine-like definition of mammalian Prx4 was accepted. The sequence of the Prx4 subfamily was highly conserved in various species. The shrimp *Mj*Prx4 showed more than 80% sequence identity to human Prx4, suggesting that they may have conserved functions. In *Drosophila*, the secretion of Prx4 protein into the hemolymph is induced by septic injury, cold, or paraquat treatment (31). In our research, the activator activity of extracellular *Mj*Prx4 protein to the JAK/STAT pathway was proved by exogenous protein injection. Obvious nuclear translocation of STAT was detected in both hemocytes and gills (**Figure 2**). Moreover, the expression of STAT-responsive AMPs was upregulated by rPrx4 injection, but not by rdmPrx4 injection (**Figure 4**). This phenomenon was abolished by Prx4 antibody blocking (**Figure 3**), indicating that the shrimp Prx4 possesses a cytokine-like activity in the anti-*Vibrio* immune response. It is worth noting that the cytokine-like activity of shrimp Prx4 is dependent on the enzyme activity conferred by the two conserved cysteines as shown in **Supplementary Figure 2**.

The quick activation and the induction of the expression of effector genes are the characteristics of innate immunity. This process is initiated by the recognition of pathogen-associated molecular patterns in the pathogen surface *via* membrane-located pattern recognition receptors. Both the Toll and JAK/STAT pathways work in recognizing pathogens, inducing the nuclear translocation of NF-κB or STAT transcription factors and the expression of the effector genes in humans and shrimp. However, the JAK/STAT pathway mainly responds to the stress process in *Drosophila* and induces the expression of stress-responsive genes, such as *Tat*A. Interestingly, the expression of *TatA* is induced in fly strains which overexpressed a high level of Prx4, and this response is not depending on the stress pressure because the expression of *pukered* gene (effector gene of JNK) in another stress response signaling is not changed (31). Moreover, the knockdown of the expression of Prx4 in flies inhibited the induced expression of *TatA* by stress pressure (septic injury, cold, or paraquat treatment), indicating a tight connection between Prx4 and the JAK/STAT pathway in *Drosophila*. In our research, this connection was proved by identifying the direct interaction of intact *Mj*Prx4 with the Domeless receptor of the JAK/STAT pathway *in vivo* and *in vitro* (**Figures 5**, **6**). The activation activity of exogenous *Mj*Prx4 to the STAT transcription factor and its downstream AMPs established a new mechanism for activation of the JAK/STAT pathway in animals.

To date, Domeless is the solely identified receptor for the JAK/STAT pathway in fruit fly (*Dm*Dome) and shrimp (*Mj*Dome). The domain prediction of two Domeless proteins in SMART software (http://smart.embl-heidelberg.de/) showed that they both contained an intracellular domain, a transmembrane domain, and an extracellular N-terminal region that contained five FN3 domains (**Supplementary Figure 3**). The sequence characteristic of the vertebrate cytokine receptor class I family is a CBM motif, which contains a set of four conserved cysteine residues in the N-terminal domain and a WSXWS motif in the C-terminus. Although *Dm*Dome and *Mj*Dome share low sequence similarity to the cytokine receptor class I family in amino acids, they both contained a set of cysteine residues. For *Dm*Dome, the cysteine residues in CBM exist in the two FN3 domains near the N-terminus (10). However, in *Mj*Dome, the cysteine residues in CBM existed in the sequence between the signal peptide and the first FN3 domain. Besides that, an additional interleukin 6 receptor (ILR) alpha domain is located in the *Mj*Dome and proved to be the interaction region for the CCD domain of a C-type lectin in shrimp. The CTLD domain of lectin binds to the bacterial surface and the hemocyte surface, thus contributing to the activation of the JAK/STAT pathway (16). In our research, the ILR domain was in the mutant Dome1-2 fragment. This fragment and the Dome1-1 fragment both contribute to the interaction of *Mj*Prx4 to *Mj*Dome (**Figure 6**). Both Dome1-1 and Dome1-2 fragments have four cysteine residues, but only Dome1-1 has the WSX motif. Since rdmPrx4 did not bind to the CBM (including Dome1-1 and Dome1-2) of *Mj*Dome (**Figure 6**) and did not have enzyme activity (**Supplementary Figure 2**), indicating that *Mj*Prx4 interacts with *Mj*Dome through disulfide linkage. Further research should be done to disclose the possibility.

In conclusion, this research reveals a novel mechanism for the activation of the JAK/STAT pathway during *Vibrio* infection in shrimp. A new ligand to the Domeless receptor was identified, and the results also expand the knowledge about the function of the Prx4 family in animals.

## Data Availability Statement

The original contributions presented in the study are included in the article/**Supplementary Material**. Further inquiries can be directed to the corresponding author.

## Author Contributions

Conceived and designed the experiments: XQR, LG, and CJK. Performed the experiments: XQR, LG, and MY. Analyzed the data and wrote the paper: XQR, LG, and CJK. All authors contributed to the article and approved the submitted version.

## Funding

The current study was supported by the National Key R&D Program of China (number 2018YFD0900303) and the National Natural Science Foundation of China (grant number 31572655).

## Conflict of Interest

The authors declare that the research was conducted in the absence of any commercial or financial relationships that could be construed as a potential conflict of interest.

## Publisher’s Note

All claims expressed in this article are solely those of the authors and do not necessarily represent those of their affiliated organizations, or those of the publisher, the editors and the reviewers. Any product that may be evaluated in this article, or claim that may be made by its manufacturer, is not guaranteed or endorsed by the publisher.

## Supplementary Material

Supplementary Figure 1 Detection of the recombinant protein and antibody of *Mj*Dorsal, *Mj*Relish, or *Mj*STAT. **(A, C, E)** Lane M, protein marker; lane 1, *E. coli* cells containing *Mj*Dorsal-pET32a, *Mj*Relish-pET30, and *Mj*STAT-pET30 without IPTG induction, total proteins; lane 2, *E. coli* cells containing *Mj*Dorsal-pET32a, *Mj*Relish-pET30, and *Mj*STAT-pET30 with IPTG induction, total proteins; lane 3, the recombinant *Mj*Dorsal, *Mj*Relish, and *Mj*STAT protein purified by His-Bind resin affinity chromatography. **(B, D, F)** Western blot detection of the Dorsal, Relish, and STAT protein in shrimp gills by using their polyclonal antibodies.

Supplementary Figure 2 Enzymatic activity detection of rPrx4 and rdmPrx4 proteins. **(A)** The purified recombinant His-tag, rPrx4-His, and rdmPrx4 proteins are expressed in *E. coli* Rosetta cells. **(B)** Peroxidase activity detection of rPrx4 and rdmPrx4 by using the method described previously (24).

Supplementary Figure 3 Sketch map of the domain architecture of Domeless proteins from *Marsupenaeus japonicus* and *Drosophila melanogaster*. The domain architecture was predicted by using SMART (http://smart.embl-heidelberg.de/).
