# Supplementary figures and images for: Peroxiredoxin 4 Interacts With Domeless and Participates in Antibacterial Immune Response Through the JAK/STAT Pathway
Source: Front Immunol. 2022 May 26;13:907183. doi: 10.3389/fimmu.2022.907183 (PMC9195186; doi:10.3389/fimmu.2022.907183)

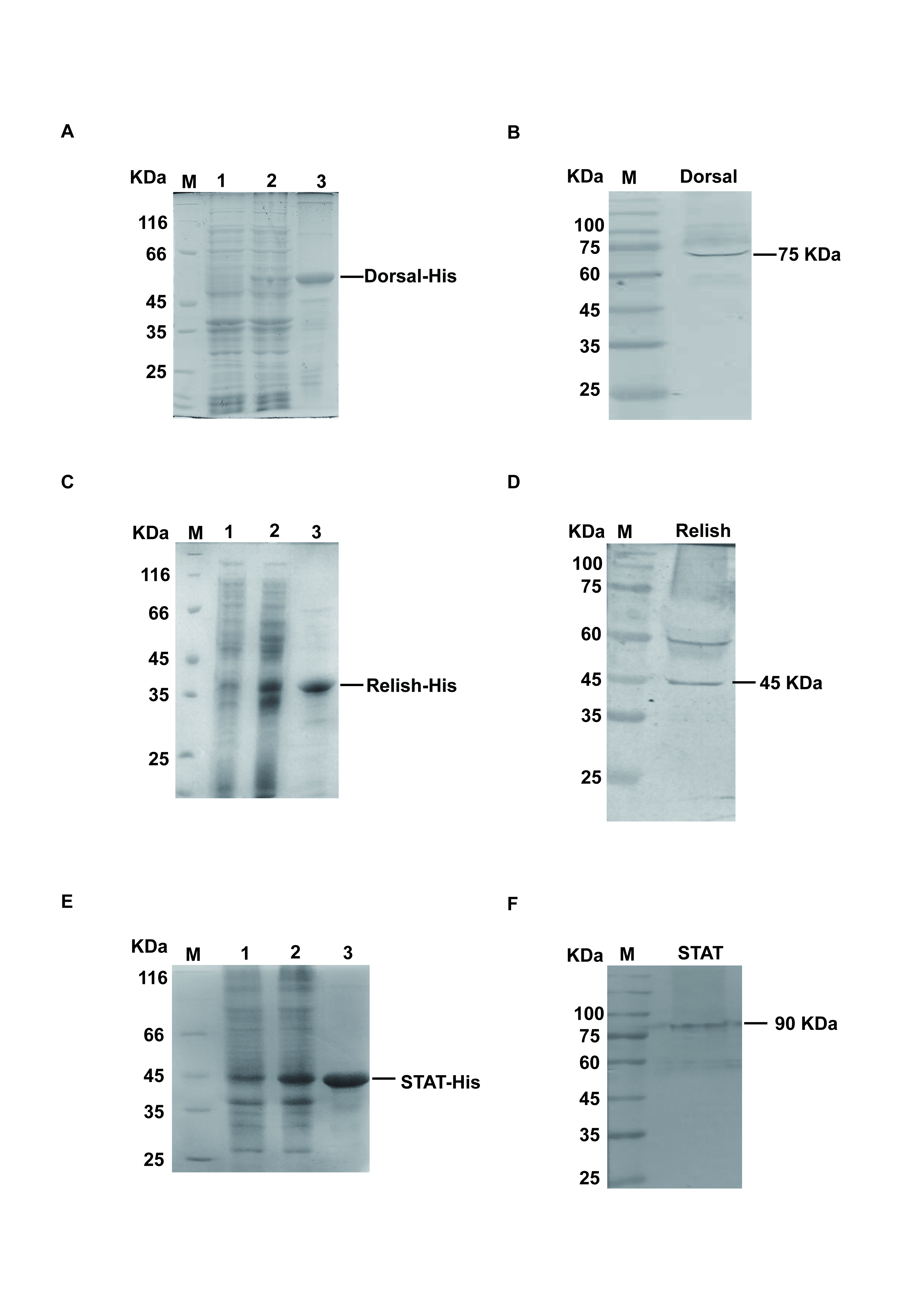

Supplement: Supplementary Figure 1 — Detection of the recombinant protein and antibody of MjDorsal, MjRelish, or MjSTAT. (A, C, E) Lane M, protein marker; lane 1, E. coli cells containing MjDorsal-pET32a, MjRelish-pET30, and MjSTAT-pET30 without IPTG induction, total proteins; lane 2, E. coli cells containing MjDorsal-pET32a, MjRelish-pET30, and MjSTAT-pET30 with IPTG induction, total proteins; lane 3, the recombinant MjDorsal, MjRelish, and MjSTAT protein purified by His-Bind resin affinity chromatography. (B, D, F) Western blot detection of the Dorsal, Relish, and STAT protein in shrimp gills by using their polyclonal antibodies. [file Image_1.tif]

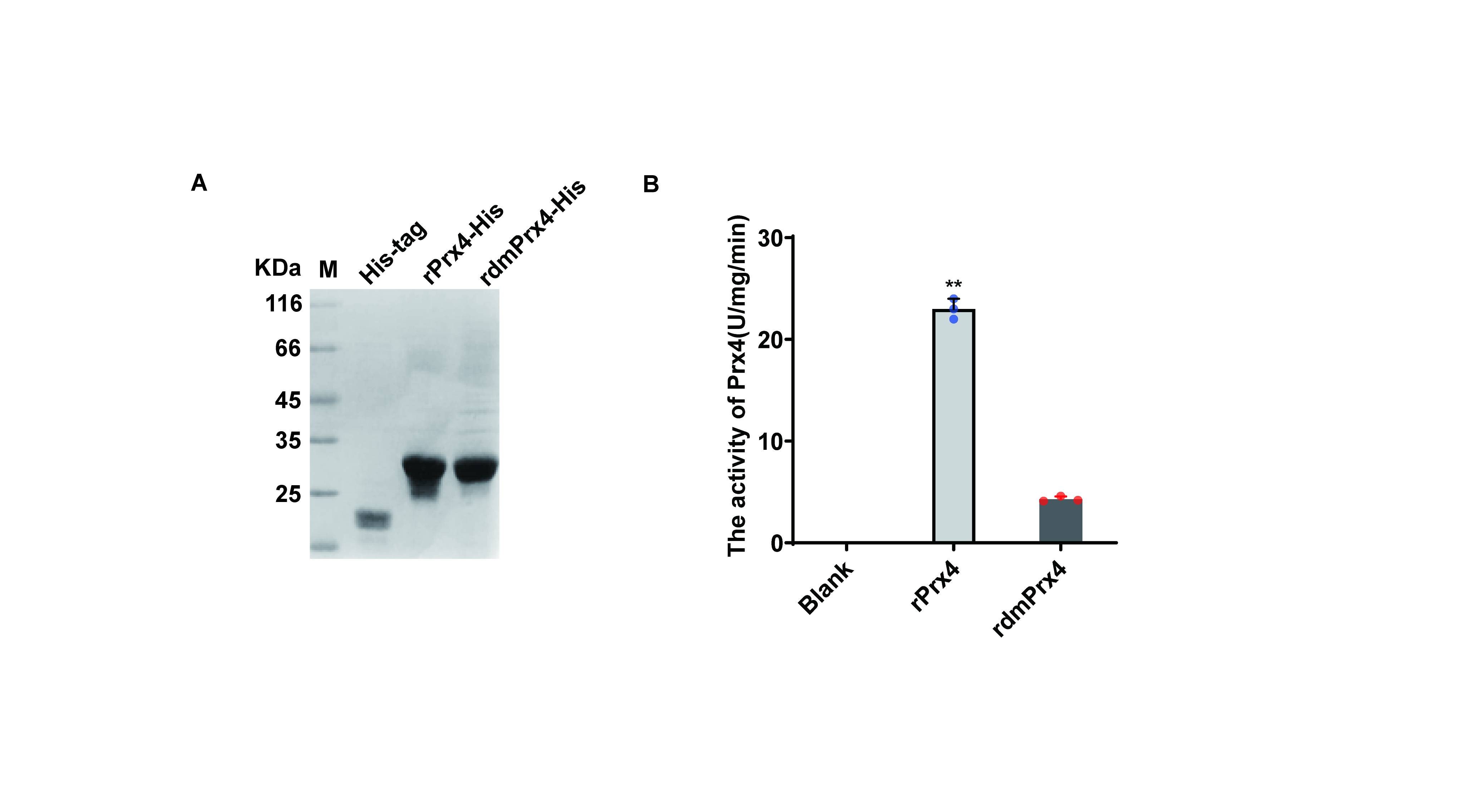

Supplement: Supplementary Figure 2 — Enzymatic activity detection of rPrx4 and rdmPrx4 proteins. (A) The purified recombinant His-tag, rPrx4-His, and rdmPrx4 proteins are expressed in E. coli Rosetta cells. (B) Peroxidase activity detection of rPrx4 and rdmPrx4 by using the method described previously (24). [file Image_2.tif]

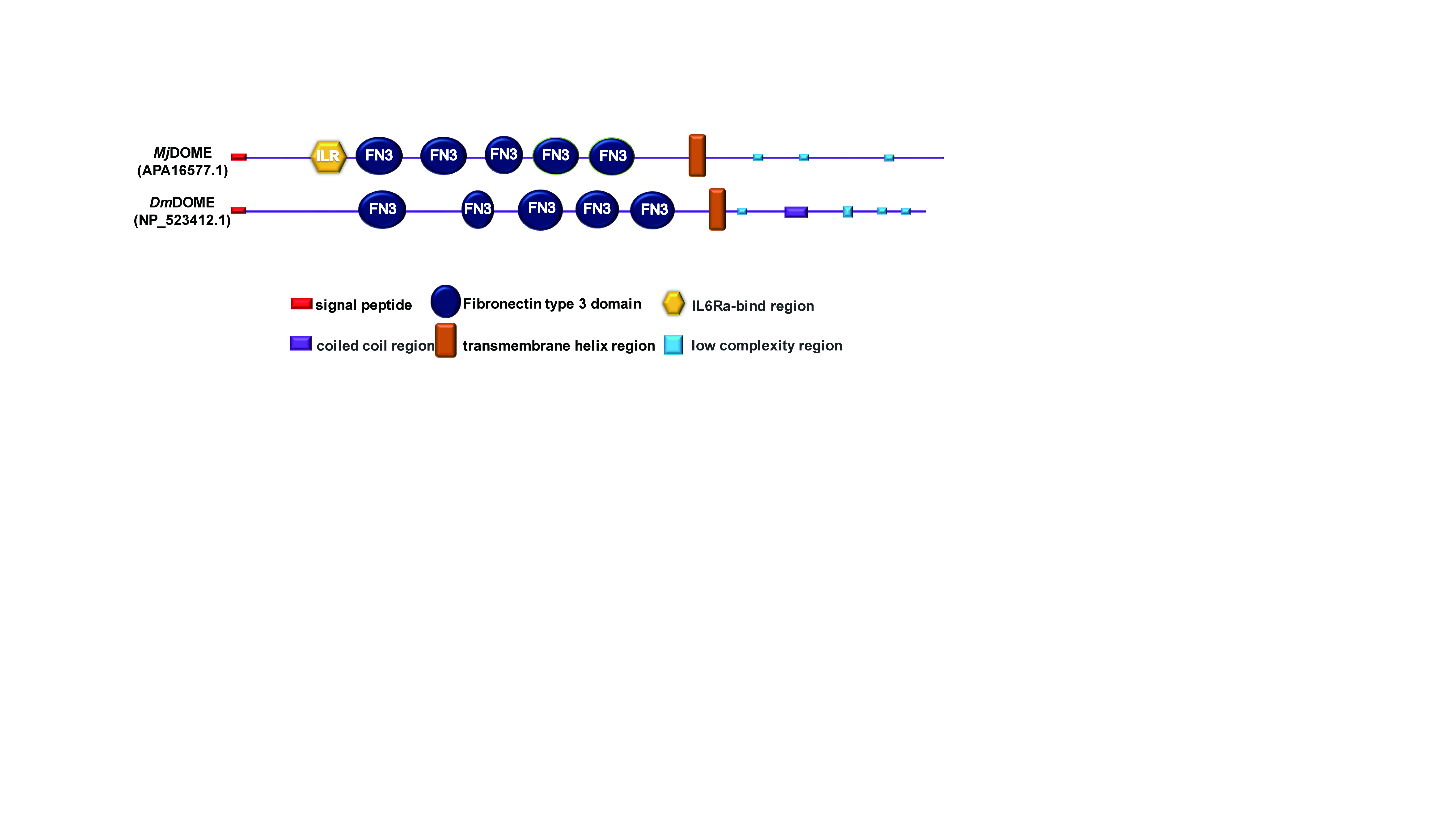

Supplement: Supplementary Figure 3 — Sketch map of the domain architecture of Domeless proteins from Marsupenaeus japonicus and Drosophila melanogaster. The domain architecture was predicted by using SMART (http://smart.embl-heidelberg.de/). [file Image_3.tif]
